# Clinically Inferred Metabolic Dysfunction-Associated Steatotic Liver Disease and Its Association with Atrial Fibrillation Subtypes: A Prospective Clinical and Cardiometabolic Analysis

**DOI:** 10.3390/life16071101

**Published:** 2026-06-30

**Authors:** Monika Różycka-Kosmalska, Boguslawa Luzak, Marcin Kosmalski

**Affiliations:** 1 Department of Electrocardiology, Medical University of Lodz, 92-213 Lodz, Poland; monika.rozycka-kosmalska@umed.lodz.pl; 2 Department of Hemostasis and Hemostatic Disorders, Medical University of Lodz, 92-215 Lodz, Poland; boguslawa.luzak@umed.lodz.pl; 3 Department of Clinical Pharmacology, Medical University of Lodz, 90-153 Lodz, Poland

**Keywords:** probable metabolic dysfunction-associated liver disease, clinically inferred MASLD, atrial fibrillation, atrial fibrillation subtypes, phenotypic heterogeneity, cardiometabolic disorder, FIB-4

## Abstract

**Background:** Metabolic dysfunction-associated steatotic liver disease (MASLD) has been linked to atrial fibrillation (AF); however, its relationship with specific AF subtypes remains unclear. This prospective, single-center, observational case–control study investigated whether MASLD is independently associated with AF presence and its subtypes. **Materials:** A total of 327 participants were analyzed, including 119 controls and 208 patients with AF. Comprehensive clinical history, anthropometric measures, laboratory testing, 24 h Holter ECG, and echocardiography were performed. Clinically inferred MASLD was defined according to the current EASL–EASD–EASO guidelines using clinical and non-invasive indices (Hepatic Steatosis Index, Fatty Liver Index, Fibrosis-4 Index). No liver biopsy or imaging confirmation of steatosis or fibrosis was performed, and therefore, the diagnosis represents a clinically inferred (“probable”) MASLD. To minimize systematic bias and improve baseline comparability between groups, propensity score matching and complementary regression analyses were applied. **Results:** Overall probable MASLD prevalence did not differ between AF and controls (42% vs. 44%, *p* = 0.742). A clear phenotypic gradient emerged across subtypes: lowest in permanent AF (PermAF, 27.1%) versus paroxysmal (47.1%) and persistent AF (51.4%) (*p* = 0.021). PermAF exhibited the most advanced comorbidity—highest CHF (78.6%), CKD (71.4%), HFpEF (48.6%), FIB-4 (median 2.67), the lowest TG/HDL–cholesterol ratio (1.93 vs. 3.32; *p* < 0.001), and progressive renal impairment. Statin therapy reached 80% in clinically inferred MASLD-positive PermAF. The elevated FIB-4 observed in PermAF must be interpreted with explicit caution: this group was substantially older (median 79.5 years) and carried the highest burden of chronic heart failure and chronic kidney disease; therefore, in this subgroup, FIB-4 most plausibly reflects age and cardio-renal comorbidity rather than histologically confirmed hepatic fibrosis. After matching, MASLD was not an independent predictor of AF presence (OR = 0.96; 95% CI: 0.59–1.46) or its clinical severity. **Conclusions:** Probable MASLD, defined by clinical and non-invasive indices, was not independently associated with AF in this cohort, but AF subtypes exhibited a clear phenotypic gradient—from a metabolically driven profile in early AF to a cardio-renal and fibrotic pattern in advanced, elderly AF. Elevated FIB-4 values in PermAF most plausibly reflect age and cardio-renal comorbidity rather than true histologically confirmed hepatic fibrosis. These findings support a phenotype- and population-dependent MASLD–AF relationship and underscore the need for imaging- and histology-verified longitudinal studies.

## 1. Introduction

Metabolic dysfunction-associated steatotic liver disease (MASLD) has emerged as the predominant chronic liver condition worldwide, representing a significant and growing public health challenge [1]. MASLD is defined by the presence of hepatic steatosis in conjunction with at least one cardiometabolic risk factor—including overweight or obesity, type 2 diabetes mellitus (T2DM), hypertension (HA), hypertriglyceridemia, or low high-density lipoprotein cholesterol concentration—in the absence of excessive alcohol consumption or other secondary causes of hepatic fat accumulation, according to the recent multisociety Delphi consensus statement [2,3]. The global prevalence of MASLD is estimated to be approximately 30–40% of the general adult population, with rates continuing to rise alongside the worldwide epidemic of obesity and metabolic syndrome [4]. In the United States alone, the burden of MASLD and its complications continues to expand, generating substantial direct healthcare costs that correlate, among other factors, with the severity of liver fibrosis as reflected by FIB-4 scores [5]. The clinical spectrum of MASLD ranges from simple hepatic steatosis to metabolic dysfunction-associated steatohepatitis (MASH), progressive hepatic fibrosis, cirrhosis, and hepatocellular carcinoma [6]. Beyond its hepatic manifestations, MASLD is increasingly recognized as a systemic metabolic disorder with profound extrahepatic consequences, particularly affecting the cardiovascular system. Cardiovascular disease represents the leading cause of death in MASLD, and the relationship between the two conditions is bidirectional, with MASLD documented in up to 75% of patients with T2DM [7]. Individuals with MASLD carry a disproportionately high burden of cardiometabolic comorbidities, and cardiovascular disease remains the leading cause of mortality in this population, surpassing liver-related complications in the majority of affected patients [8]. Indeed, MASLD has been shown to adversely affect cardiovascular structure and function, promote accelerated atherosclerosis, and increase the risk of heart failure, with heart failure with preserved ejection fraction constituting the dominant HF phenotype in MASLD cohorts, underscoring the critical importance of a holistic, multidisciplinary approach to its management [9]. Among the cardiovascular complications associated with MASLD, atrial fibrillation (AF)—the most common sustained cardiac arrhythmia in clinical practice—has attracted growing scientific interest. AF is conventionally classified into paroxysmal (PAF), persistent (PeAF), and permanent (PermAF) forms, each differing in its natural history, pathophysiological substrate, and therapeutic implications. The pathophysiological link between MASLD and AF is complex and multifactorial, encompassing shared upstream mechanisms, such as insulin resistance, chronic low-grade inflammation, oxidative stress, sympathetic nervous system activation [10], and ectopic adipose tissue deposition around the heart [11]. These mechanisms may collectively promote atrial structural and electrical remodeling, thereby creating a substrate favorable for the initiation and perpetuation of AF [10]. Accumulating evidence suggests that MASLD is independently associated with an increased long-term risk of incident AF, even after adjustment for conventional cardiovascular risk factors [11]. A recent updated systematic review and meta-analysis confirmed that MASLD confers a significantly elevated risk of AF development, reinforcing the notion that the liver-heart axis represents a clinically relevant pathophysiological pathway [12]. Moreover, the interplay between MASLD and AF appears bidirectional, as AF may itself exacerbate hepatic congestion and metabolic dysfunction through hemodynamic alterations and neurohormonal activation [13,14]. Although these subtypes are commonly arranged into a clinical sequence, the cross-sectional design of most available evidence precludes inference about true temporal progression from one subtype to another. Furthermore, the extent to which cardiometabolic comorbidities, heart failure phenotype, renal dysfunction, and concomitant medication use modulate the MASLD–AF relationship remains insufficiently defined. This caveat is particularly important because non-invasive hepatic fibrosis indices—most notably FIB-4, derived from age, aspartate aminotransferase, alanine aminotransferase, and platelet count—are intrinsically age-dependent and may also be influenced by systemic illness, cardio-hepatic congestion, and pharmacotherapy. Consequently, elevated FIB-4 values in elderly, cardio-renally-impaired AF populations should not be interpreted as direct evidence of histologically confirmed hepatic fibrosis but rather as a composite surrogate marker of age, systemic illness, and cardio-renal dysfunction [13,15,16].

The aim of the present study was, therefore, to investigate the association between clinically inferred MASLD and different types of AF in a prospectively enrolled observational cohort, providing novel insights into the hepato-cardiac relationship and its potential implications for clinical risk stratification and patient management. Consistent with the observational design, our analysis focused on identifying phenotype-specific associations and accounting for potential confounders, such as age and chronic comorbidities.

## 2. Materials and Methods

### 2.1. Characteristics of Patients

This single-center, observational case–control study was conducted and reported in accordance with the Strengthening the Reporting of Observational Studies in Epidemiology guidelines. The study was guided by a pre-specified written protocol developed prior to subject enrollment, which delineated the primary and secondary objectives, inclusion/exclusion criteria, definitions of clinical variables, and a pre-defined statistical analysis plan, including propensity score matching and complementary regression modeling. The protocol, together with all study materials, was approved by the Bioethics Committee of the Medical University of Lodz (approval number RNN/01/24, dated 13 February 2024) prior to the initiation of recruitment. The study was conducted in accordance with the Declaration of Helsinki.

A total of 327 patients (114 women and 213 men) with a mean age of 66.5 ± 15.1 years were prospectively enrolled between February 2024 and March 2026 at the Department of Electrocardiology, Medical University of Lodz, during hospitalization for various internal medicine conditions. General inclusion criteria were as follows: age over 18 years, provision of written informed consent to participate in the study, and preserved verbal and logical communication enabling reliable contact with the patient.

Participants were allocated to one of the following groups:

- Control group (*n* = 119): individuals with no documented history of AF.
- AF + group (*n* = 208): individuals with confirmed AF, subclassified into
  ○ paroxysmal AF (PAF, *n* = 68);
  ○ persistent AF (PeAF, *n* = 70);
  ○ permanent AF (PermAF, *n* = 70).

**Figure 1:**
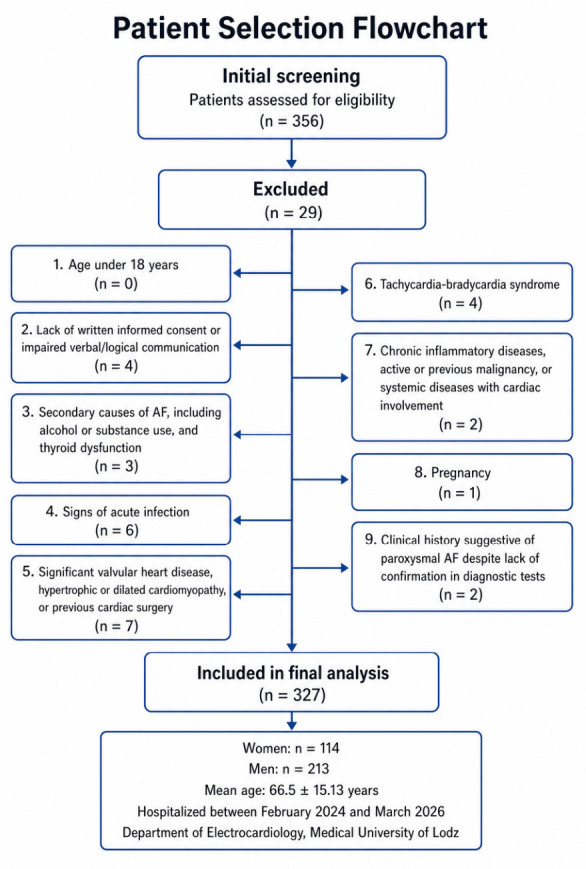
Flowchart of patient selection.

The detailed screening process and complete exclusion criteria are illustrated in the patient selection flowchart (Figure 1).

Twenty-nine patients were excluded based on the predefined criteria: lack of written informed consent or impaired verbal/logical communication (*n* = 4), secondary causes of atrial fibrillation (AF) including alcohol or substance use and thyroid dysfunction (*n* = 3), signs of acute infection (*n* = 6), significant valvular heart disease, hypertrophic or dilated cardiomyopathy, or previous cardiac surgery (*n* = 7), tachycardia–bradycardia syndrome (*n* = 4), chronic inflammatory diseases, active or previous malignancy, or systemic diseases with cardiac involvement (*n* = 2), pregnancy (*n* = 1), and a clinical history suggestive of paroxysmal AF despite lack of diagnostic confirmation (*n* = 2). No patients were excluded due to age under 18 years (*n* = 0). For each patient, a detailed medical history and physical examination were performed. Chronic heart failure (CHF) was defined and classified based on the left ventricular ejection fraction (LVEF) into three categories: heart failure with reduced ejection fraction (HFrEF, LVEF ≤ 40%), heart failure with mildly reduced ejection fraction (HFmrEF, LVEF 41–49%), and heart failure with preserved ejection fraction (HFpEF, LVEF ≥ 50%). Anthropometric measurements included body weight, height, waist circumference, and hip circumference, which were used to calculate body mass index (BMI) and waist-to-hip ratio (WHR). The analysis also accounted for the potential influence of pharmacological treatments, including medications for diabetes, dyslipidemia (e.g., statins), and electrolyte imbalances (e.g., potassium-sparing diuretics) on the investigated relationships.

Subsequently, a fasting venous blood sample was collected to assess complete blood count, glucose concentration, glycated hemoglobin (HbA1c), alanine aminotransferase (ALT), aspartate aminotransferase (AST), gamma-glutamyltransferase (GGTP), lipid profile, creatinine, urea, and uric acid. The estimated glomerular filtration rate (eGFR) was calculated using the Modification of Diet in Renal Disease (MDRD) equation [17]. All patients then underwent standard electrocardiographic assessment, including 24 h Holter ECG monitoring, and transthoracic echocardiography was also performed to assess LVEF. Each patient was assessed for the presence of MASLD. Clinically inferred MASLD was defined according to the current EASL–EASD–EASO guidance using clinical and non-invasive indices of hepatic steatosis and fibrosis, including the Hepatic Steatosis Index, Fatty Liver Index, and Fibrosis-4 Index [18]. No liver ultrasound, transient elastography, controlled attenuation parameter, or histological assessment was performed to confirm the diagnosis; the classification therefore represents a clinically inferred—and not an imaging- or histology-verified—MASLD. In addition, three non-invasive continuous indices were calculated:

- The Hepatic Steatosis Index (HSI)—a serum-based marker correlated with ultrasonographic steatosis;
- Fatty Liver Index (FLI)—a validated surrogate widely used in cardiometabolic cohorts;

Fibrosis-4 Index (FIB-4)—a non-invasive proxy of hepatic fibrosis derived from age, AST, ALT, and platelet count. These indices were selected because liver biopsy—albeit the diagnostic gold standard for MASLD—carries recognized limitations in this setting [19]. FIB-4 was analyzed as a composite, non-invasive continuous biomarker. Because FIB-4 incorporates age, AST, ALT, and platelet count, it is intrinsically age-dependent and may also be influenced by systemic illness, renal dysfunction, cardio-hepatic congestion, and pharmacotherapy. Consequently, elevated FIB-4 values in our cohort—particularly in elderly patients with chronic heart failure and chronic kidney disease—are not interpreted as direct evidence of histologically confirmed hepatic fibrosis, but rather as a composite marker influenced by age and cardio-renal comorbidity.

### 2.2. Statistical Analysis

The normality of the distribution of the analyzed variables was assessed using the Shapiro–Wilk test. *p*-values were reported with three significant digits (e.g., *p* < 0.001). Descriptive statistics were presented as mean ± standard deviation (SD) for normally distributed continuous variables or as median with interquartile range (IQR; Q1; Q3) for non-normally distributed variables. Additionally, the standardized mean difference (SMD) was calculated and reported in tables as absolute values. Categorical variables were expressed as numbers and percentages. Percentages were standardized to one decimal place.

For normally distributed variables, differences between two groups (AF vs. control) were analyzed using the unpaired Student’s *t*-test, whereas comparisons among more than two groups (AF subtypes and control) were performed using one-way analysis of variance (ANOVA) followed by Tukey’s post hoc test for unequal sample sizes.

For variables that did not follow a normal distribution, the Mann–Whitney *U* test was used for comparisons between two groups, while the Kruskal–Wallis test followed by Dunn’s multiple comparison test was applied for comparisons among more than two groups. Categorical variables were compared using the χ^2^ test or Fisher’s exact test, as appropriate. All statistical analyses were performed using STATISTICA software (Dell Inc., Round Rock, TX, USA, 2016; Dell Statistica, Data Analysis System, version 13 (software.dell.com, accessed between 1 March and 30 April 2026)). A two-tailed *p*-value < 0.05 was considered statistically significant.

Propensity score matching (PSM) was applied to reduce the imbalance between patients with and without MASLD. Propensity scores were estimated using logistic regression, and patients were matched in a 1:1 nearest-neighbor manner. The propensity score included the following pre-specified covariates: age, sex, waist–hip ratio, presence of T2DM, presence of hypertension, total cholesterol, HDL cholesterol, LDL cholesterol, triglycerides, bilirubin, creatinine, and estimated glomerular filtration rate (eGFR). Variables closely related to the metabolic phenotype or diagnosis of MASLD (e.g., BMI, TG, liver enzymes: ALT, AST, GGTP, and steatosis indices: FLI, HSI) were excluded to avoid overadjustment. Covariate balance before and after matching was assessed using standardized mean differences. The final matched cohort consisted of 115 matched pairs. These statistical analyses were performed using R software R software version 4.5.2 (2025-10-31 ucrt)) (R Foundation for Statistical Computing, Vienna, Austria). The association between MASLD and the presence of atrial fibrillation (AF) was evaluated using logistic regression models. To account for the matched-pair design, conditional logistic regression was additionally performed. AF severity, categorized as no AF, paroxysmal AF, persistent AF, and permanent AF, was analyzed using ordinal logistic regression. Exploratory analyses were also conducted using multinomial logistic regression. The proportional odds assumption for ordinal regression was formally tested and confirmed. All analyses were conducted using reproducible statistical scripts.

## 3. Results

In total, 327 participants, comprising 119 controls and 208 patients with AF, were included in the analysis. AF patients had a significantly higher prevalence of chronic heart failure (CHF) than controls (61% vs. 32%; *p* < 0.0001) (Table 1). Among participants with clinically inferred MASLD, HFmrEF was observed more frequently. Chronic kidney disease (CKD) occurred significantly more often in AF patients than in the control group (*p* < 0.001). There were no significant differences between groups in the prevalence of coronary artery disease (CAD), HA, T2DM, and MASLD. Men were more numerous in the AF group than in the control group, although this difference was not statistically significant (69% vs. 60%, *p* = 0.096). AF patients were significantly older than controls (*p* < 0.001). No significant differences were found between the AF and control groups with respect to BMI, WC, WHR, uric acid, ALT, AST, GGTP, FPG, HbA1c, EF, HSI, or FLI. However, AF patients exhibited significantly different lipid and renal profiles, with lower total cholesterol (T-CH), LDL cholesterol (LDL-CH), triglycerides (TG), and eGFR, and higher HDL cholesterol (HDL-CH), creatinine, and urea concentrations compared with controls (all *p* < 0.05). The FIB-4 index was also significantly higher in the AF group than in controls (*p* < 0.001).

When AF was stratified by subtype, CHF remained significantly more frequent in all AF subgroups than in the control group (*p* < 0.001), with the highest prevalence in PermAF (78.6%) (Table 2). Across the CHF phenotypes, a systematic shift was observed: HFrEF was least frequent in PermAF (8.6%), whereas HFpEF predominated (48.6%, *p* < 0.001) and HFmrEF reached its peak prevalence (21.4%, *p* = 0.001). MASLD prevalence differed across AF subtypes (*p* = 0.021). The lowest MASLD prevalence was found in PermAF (27.1%), which was significantly lower than in the control (43.7%, *p* < 0.05), PAF (47.1%, *p* < 0.05), and PeAF (51.4%, *p* < 0.05) groups. The proportions of MASLD in the control, PAF, PeAF, and PermAF groups were therefore 44%, 47%, 51%, and 27%, respectively. CKD was most prevalent in PermAF (71.4%) compared with controls (29.4%, *p* < 0.001), PAF (35.3%, *p* < 0.001), and PeAF (50.0%, *p* < 0.05), consistent with progressive renal impairment across AF categories. TG/HDL ratio was lowest in PermAF and significantly differed from controls, PAF, and PeAF (all *p* < 0.05). TG concentrations were also lowest in PermAF and differed significantly from all three reference groups. In contrast, T-CH differed across subtypes (overall *p* = 0.003), but only the pairwise comparison of PermAF vs. PAF reached statistical significance. Age increased progressively across AF subtypes, from PAF (65.0 [56.5; 72.0]) to PeAF (70.0 [65; 75]) to PermAF (79.5 [73; 85]) (overall *p* < 0.001; SMD = 1.40). The HSI was significantly lower in PermAF than in PAF and PeAF (*p* < 0.05 for both) but did not differ significantly from controls. The FIB-4 index was significantly higher in PermAF (2.67 [2.0; 3.6]) than in controls, PAF, and PeAF (all *p* < 0.05). However, FIB-4 should be interpreted cautiously in this cohort because the PermAF group was older and had a higher burden of chronic heart failure and chronic kidney disease; therefore, elevated FIB-4 may reflect age and cardio-renal comorbidity rather than histologically confirmed hepatic fibrosis.

Analysis of MASLD prevalence in patients at increased risk of AF (the groups of patients with CAD, CHF, HA, T2DM, CKD, EF ≤ 50%, or BMI ≥ 30 kg/m^2^) showed no significant association with sex, CAD, HA, EF ≤ 50%, or obesity between the control and AF groups. Within patients with CKD, clinically inferred MASLD prevalence showed a numerical trend toward lower values in the AF + group than in controls (28.4% vs. 45.7%, *p* = 0.065), although this difference did not reach statistical significance. By contrast, clinically inferred MASLD prevalence was significantly lower in AF + patients with CHF than in the control group with CHF (43.3% vs. 60.5%, *p* = 0.046), and it was also significantly lower in AF + patients with T2DM than in controls with T2DM (48.7% vs. 73.2%, *p* = 0.008) (Table 3).

In the analysis of clinically inferred MASLD prevalence according to AF subtype, significant differences were observed for sex distribution and CHF (Table 4). Among men, clinically inferred MASLD prevalence differed across groups, with the lowest frequency observed in PermAF (21%) compared with the control group (52%) (*p* < 0.01) and the other AF subtypes (PAF vs. PermAF *p* < 0.05; PermAF vs. PeAF *p* < 0.001). Among women, MASLD prevalence did not differ significantly across groups (*p* = 0.240). CHF was also significantly associated with clinically inferred MASLD prevalence across AF categories (*p* = 0.023), with the lowest MASLD frequency observed in PermAF (PermAF vs. PeAF *p* < 0.05). Within HF phenotypes, clinically inferred MASLD prevalence differed across groups among HFrEF patients (overall *p* = 0.044; 65.2%, 38.5%, 46.7%, 33.3% in control, PAF, PeAF, and PermAF, respectively). In contrast, clinically inferred MASLD prevalence did not differ significantly across groups among HFmrEF patients (*p* = 0.450) or HFpEF patients (*p* = 0.280). For hypertension, T2DM, obesity (BMI ≥ 30 kg/m^2^), and CKD, clinically inferred MASLD prevalence showed a consistent numerical pattern of being lowest in the PermAF group (31.9%, 39.1%, 54.5%, and 22.0%, respectively), but none of these differences reached statistical significance after correction for multiple comparisons (overall *p* = 0.076, 0.051, 0.073, and 0.148, respectively). CAD and reduced ejection fraction were not associated with clinically inferred MASLD prevalence in any AF subtype (*p* > 0.6 for both).

Table 5 summarizes the medication data, showing that the use of cardiovascular and antidiabetic medications differed across AF subtypes and according to clinically inferred MASLD status. In all study groups, patients with clinically inferred MASLD were more frequently treated with renin-angiotensin system blockers, diuretics, aldosterone antagonists, statins, and glucose-lowering agents than those without clinically inferred MASLD. The highest proportions of ACE, loop diuretic, statin, metformin, and SGLT2 inhibitor use were observed in the clinically inferred MASLD-positive groups, particularly among patients with permanent and persistent AF.

To further assess the potential association between clinically inferred MASLD and the presence and subtypes of AF, additional statistical analyses were performed to minimize the impact of potential confounding factors. Because patients with clinically inferred MASLD differed from those without MASLD in several clinical and metabolic characteristics, propensity score matching (PSM) was applied to create more comparable groups. The propensity score was defined as the probability of clinically inferred MASLD conditional on clinically relevant covariates, including age, sex, WHR, T2DM, HA, lipid profile, and kidney function. Variables closely related to the metabolic phenotype or diagnosis of clinically inferred MASLD (e.g., BMI, TG, liver enzymes, and steatosis indices) were excluded to avoid overadjustment. Before matching, a substantial imbalance between groups was observed, particularly for WHR (SMD ≈ 0.63), HA (SMD ≈ 0.53), and T2DM (SMD ≈ 0.46). After matching, covariate balance improved markedly, with most standardized mean differences (SMD) below 0.1 and a maximum of approximately 0.13, indicating good comparability. The final matched sample included 115 patient pairs. The association between MASLD and AF presence was evaluated using logistic regression, followed by conditional logistic regression accounting for the matched design. AF severity was analyzed using ordinal logistic regression, with AF categorized into four stages: no AF, paroxysmal, persistent, and permanent AF. The proportional odds assumption was satisfied. Across all models, the estimated effects were consistent but not statistically significant. In logistic regression, clinically inferred MASLD was associated with an odds ratio (OR) of 0.96 (95% CI: 0.59–1.46) for AF presence, while conditional logistic regression yielded similar OR values. Similar results were found in the ordinal model, suggesting a non-significant tendency toward lower odds of more advanced AF stages. Additional analyses, including multinomial models and analyses restricted to patients with AF, produced consistent results, with no statistically significant associations observed. Overall, despite the use of PSM and multiple complementary modeling approaches, no significant association was found between clinically inferred MASLD and either AF presence or severity. These results suggest that MASLD is not an independent predictor of AF in this cohort. Limitations include the observational design, potential residual confounding, moderate sample size after matching, and the cross-sectional assessment of AF severity.

Considering the overall findings, including comorbidities and clinical characteristics, distinct profiles can be delineated for different types of AF.

**Figure 2:**
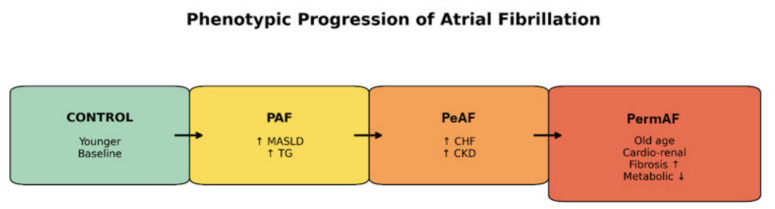
Phenotypic progression and remodeling across atrial fibrillation subtypes. PAF—paroxysmal atrial fibrillation; PeAF—persistent atrial fibrillation; PermAF—permanent atrial fibrillation; CKD—chronic kidney disease; CHF—chronic heart failure; MASLD—clinically inferred metabolic dysfunction-associated steatotic liver disease; TG—triglycerides. Elevated FIB-4 in PermAF is interpreted as a composite marker of age and cardio-renal comorbidity, not as a direct indicator of histologically confirmed hepatic fibrosis.

Figure 2 illustrates the progression of AF from control subjects through PAF and PeAF to PermAF, highlighting the associated phenotypic transformation.

Early stages of AF are characterized by a metabolic phenotype, reflected by a higher prevalence of clinically inferred MASLD and elevated TG. With the progression of the disease, a transition toward a cardio-renal phenotype is observed, which is marked by increasing prevalence of CHF and CKD, along with progressive deterioration of renal function.

In the most advanced stage (PermAF), the phenotype shifts further toward a fibrotic and organ dysfunction pattern, characterized by increased fibrosis markers. Age emerges as a key determinant of this progression.

## 4. Discussion

In the present prospective, single-center, observational case–control study, clinically inferred MASLD was not independently associated with AF presence or severity after rigorous adjustment for cardiometabolic, renal, and treatment-related confounders. Nevertheless, AF subtypes exhibited clinically meaningful phenotypic heterogeneity: PAF was characterized by the highest prevalence of clinically inferred MASLD and the most unfavorable lipid profile, PeAF showed intermediate cardiometabolic and cardio-renal features, and PermAF was dominated by advanced age (median 79.5 years), CHF (78.6%), CKD (71.4%), the lowest proportion of HFrEF (8.6%), and the highest proportions of HFpEF (48.6%) and HFmrEF (21.4%). Collectively, these findings reinforce the concept of AF as a dynamic, progressive disease defined by a continuum from modifiable cardiometabolic risk factors to irreversible structural remodeling and permanent arrhythmia [20].

### 4.1. Aging as a Key Determinant of the Phenotypic Gradient

Aging emerged as a central determinant of the phenotypic gradient observed across AF subtypes, with median age increasing progressively from 65 years in PAF to 70 years in PeAF and 79.5 years in PermAF (max |SMD| = 1.40). This pattern is consistent with epidemiological data indicating that AF prevalence increases sharply with advancing age, reaching 21.9% in patients over 85 years of age in primary-care cohorts [21]. Aging contributes to the AF substrate not only through cumulative exposure to cardiovascular risk factors but also via intrinsic structural and electrophysiological remodeling—including fibrosis, conduction abnormalities, and mechanical dysfunction—that together define the spectrum of atrial myopathy [22,23].

### 4.2. The Cardio-Renal Continuum

The present findings provide additional support for a cardio-renal continuum underlying AF progression. Renal function deteriorated systematically across AF categories, paralleled by a steady rise in CKD prevalence. AF and CKD are closely interconnected through shared mechanisms including inflammation, oxidative stress, sympathetic and renin–angiotensin–aldosterone system activation, and structural atrial remodeling, with bidirectional causality in which each condition promotes the progression of the other [24,25]. Importantly, this cardio-renal axis was mirrored by the rising prevalence of CHF and the shift in HF-phenotype distribution across AF categories, with HFpEF predominating in PermAF—consistent with the recognized role of HFpEF as a common final substrate linking hepatic congestion, atrial remodeling, and advanced AF [1,26]. Together, these findings integrate well with the contemporary staging model of AF, in which each phenotype reflects a different weighting of metabolic, cardio-renal, and structural drivers [27,28].

### 4.3. The Apparent “Metabolic Paradox” in Advanced AF—Linking TG/HDL Ratio to Metabolic Phenotype

Another important observation is the progressive reduction in metabolic features along the AF-subtype gradient: TG decreased from 144 mg/dL (controls) to 133, 118, and 88 mg/dL; the TG/HDL cholesterol ratio was 3.32, 3.04, 3.11, and 1.93 (overall *p* < 0.001). The clinically inferred MASLD prevalence was 43.7%, 47.1%, 51.4%, and 27.1% (*p* = 0.021) across groups. The metabolic gradient described here refers throughout to clinically inferred MASLD defined by clinical and non-invasive indices; as no imaging- or histology-based MASLD verification was performed, this gradient relates to clinically defined phenotypes rather than to biopsy-proven steatosis or steatohepatitis.

First, MASLD has been consistently associated with an increased long-term risk of incident AF, even after adjustment for conventional cardiovascular risk factors, and the most recent updated meta-analysis confirmed this association [12]. In advanced AF, the disease trajectory may instead shift from predominantly metabolic drivers toward organ damage and fibrosis dominance, attenuating the overt metabolic signature [27].

Second, simple lipid-derived indices—and the TG/HDL cholesterol ratio in particular—deserve specific attention in this context. Recent evidence indicates that an elevated TG/HDL ratio is specifically associated with an increased risk of ischemic stroke in patients with paroxysmal AF, supporting its role as a simple, widely available marker of a metabolically high-risk AF phenotype [29]. In our cohort, TG/HDL paralleled clinically inferred MASLD prevalence and absolute TG concentrations across AF categories—highest in PAF, lowest in PermAF—consistent with the concept that a metabolically unfavorable lipid phenotype predominates in earlier, still-modifiable AF stages and declines as the disease shifts toward cardio-renal and fibrotic determinants.

Third, the reduction in circulating lipids is likely amplified by pharmacological modification: 80% of MASLD-positive patients in the PermAF group received statin therapy (compared with 59% in MASLD-positive PAF and 73% in MASLD-positive controls), which may account for the lower LDL-cholesterol, TCH, and TG concentrations observed in advanced AF. Fourth, the metabolic demands and cachectic tendency of advanced CHF may further lower circulating TG independently of hepatic status. Finally, diuretics, SGLT2 inhibitors, and other glucose-lowering agents may also modulate circulating lipid and metabolic markers and should therefore be considered when interpreting cross-sectional metabolic gradients across AF subtypes [10].

Taken together, the “metabolic paradox” in advanced AF should be interpreted as a stage- and population-dependent phenotypic transition—rather than as evidence of a true inverse biological relationship between MASLD and AF—and reflects the convergence of disease-stage progression, pharmacotherapy, and the systemic consequences of advanced comorbidity.

### 4.4. FIB-4, Inflammation, and the Fibrotic Switch—Interpretation in Elderly AF Populations

The FIB-4 index was significantly higher in PermAF than in controls, PAF, and PeAF, reaching the conventional threshold suggestive of advanced hepatic fibrosis (>2.67) in a substantial proportion of patients. However, this finding must be interpreted with explicit caution and cannot be regarded as evidence of histologically confirmed hepatic fibrosis in this cohort. Several converging considerations underline this caveat.

Recent evidence positions FIB-4 as a prognostic marker of cardiovascular outcomes and AF severity, reflecting systemic fibrotic burden rather than purely hepatic fibrosis [15,30]. The FIB-4 signal observed in our cohort should, however, be interpreted within the broader framework of inflammatory and fibrotic biomarkers that link metabolic dysfunction to atrial remodeling. Recent narrative syntheses have systematically integrated inflammatory cytokines, profibrotic mediators (including transforming growth factor-β and galectin-3), extracellular matrix turnover markers, and imaging-based surrogates of atrial fibrosis (cardiac magnetic resonance T1 mapping, late gadolinium enhancement quantification) into a continuum that bridges metabolic syndrome, atrial cardiomyopathy, and AF [31,32]. Importantly, this integrative perspective emphasizes that elevated circulating inflammatory and fibrotic biomarkers—even in the absence of overt hepatic disease—reflect a systemic profibrotic milieu that promotes atrial structural remodeling and conduction heterogeneity, providing a mechanistic rationale for considering FIB-4, together with such biomarkers, rather than as a stand-alone hepatic marker [33,34]. From a practical standpoint, the apparent “fibrotic switch” observed in PermAF in our analysis should therefore be interpreted not as a specific hepatic phenomenon but as a surrogate of systemic fibrotic and inflammatory burden shared between the liver and the atrial myocardium.

Nevertheless, elevated FIB-4 values in elderly, cardio-renally-impaired AF populations require particularly cautious interpretation. First, FIB-4 is derived from age, AST, ALT, and platelet count, and its values are intrinsically age-dependent: in age-stratified analyses, upper FIB-4 cut-offs rise substantially with age quartiles (from 1.72 in the lowest to 4.20 in the highest quartile), which may compromise specificity in older populations [16]. In our cohort, the median age in PermAF was 79.5 years, and a substantial proportion of FIB-4 elevation in this subgroup may therefore reflect age-related effects rather than true hepatic fibrosis. Second, in a large AF-specific cohort (>10,500 patients), high FIB-4 (>2.67) was associated with increased risk of major bleeding and heart failure hospitalization, leading the authors to conclude that in AF populations, FIB-4 may reflect venous congestion and systemic illness rather than hepatic fibrosis itself [35]. Third, similar conclusions have been drawn from population-based imaging studies, in which liver stiffness—but not fatty liver disease—was associated with prevalent AF, with the association largely explained by congestive hepatic changes and reverse cardio-hepatic causality [13]. Consequently, the elevated FIB-4 values observed in our PermAF subgroup should be regarded as a composite surrogate marker of systemic illness, hepatic congestion, age-related effects, and cardio-renal dysfunction rather than as direct evidence of histologically confirmed liver fibrosis, the latter remaining the reference standard for any meaningful fibrotic assessment [19].

### 4.5. Fibrosis, Atrial Cardiomyopathy, and Integrative Interpretation

Atrial fibrosis itself plays a central role in current models of AF pathophysiology, contributing to both arrhythmia initiation and maintenance via impaired conduction, conduction heterogeneity, and promotion of re-entrant circuits [26,34]. Recent mechanistic and clinical reviews underscore that the same systemic and paracrine profibrotic mechanisms that remodel the liver can promote structural remodeling of the heart and contribute to atrial cardiomyopathy [27,33]. The present findings integrate well with this evolving conceptual framework, which reconceptualizes AF not merely as an electrical disorder but as a manifestation of multisystem fibrotic remodeling, encompassing metabolic, inflammatory, and cardio-renal drivers [22,27,33].

### 4.6. Clinical Implications

These findings have several implications for clinical practice. First, clinically inferred MASLD and AF should be jointly assessed, recognizing that in everyday clinical practice most MASLD diagnoses are clinically inferred and based on clinical and non-invasive indices, and that their relationship is phenotype-dependent, population-dependent, and modulated by age, heart failure, renal dysfunction, lipid phenotype, and pharmacotherapy. Second, simple laboratory measures such as the TG/HDL cholesterol ratio may serve as clinically useful adjuncts for phenotype-specific risk stratification across the AF continuum—particularly because they can be derived from routine lipid panels without additional imaging or specialized scoring [29]. Third, the high FIB-4 values observed in advanced AF should not be interpreted in isolation as evidence of histologically confirmed hepatic fibrosis, but rather as a composite marker of systemic illness, age-related effects, and cardio-renal dysfunction. In our cohort, the elevated FIB-4 in PermAF most plausibly reflects age and cardio-renal comorbidity rather than true hepatic fibrosis, and particularly in elderly populations, FIB-4 values require cautious interpretation [16,34]. Fourth, the apparent “metabolic paradox” in advanced AF should not be misread as evidence of a protective effect of metabolic dysfunction, but as a marker of disease-stage transition that may carry prognostic significance independent of the underlying metabolic status. Early identification and management of metabolic dysfunction—including MASLD screening and aggressive control of cardiometabolic risk factors—may be particularly valuable for preventing AF initiation and paroxysmal-to-persistent progression [20,36]; in advanced AF, attention should instead shift toward cardio-renal optimization and integrated multidisciplinary care [37].

### 4.7. Future Research Directions

Future research should address several unresolved questions highlighted by the present analysis. First, longitudinal studies incorporating imaging- or histology-verified MASLD assessment are needed to determine whether the apparent metabolic-to-cardio-renal transition across AF subtypes reflects true disease progression or a survivor/treatment bias. Second, the prognostic value of serial FIB-4 monitoring in AF populations requires validation in cohorts with concurrent imaging-based (transient elastography, MR elastography) or histological confirmation of hepatic fibrosis and with explicit assessment of hepatic congestion and renal function. Until such data are available, FIB-4 in AF populations should remain interpreted as a composite marker of systemic illness rather than as a direct hepatic fibrosis biomarker [37,38]. Third, the precise contribution of the TG/HDL ratio and other simple lipid-derived indices to phenotype-specific risk stratification across AF subtypes warrants dedicated prospective investigation [29,31]. Finally, future studies should adopt pre-specified analytical protocols, STROBE-compliant reporting, more detailed propensity score and inverse probability of treatment weighting methodology with explicit specification of covariates and matching algorithms, and standardized definitions of CHF phenotypes in order to improve the comparability of findings across cohorts investigating the MASLD–AF relationship.

### 4.8. Limitations

Several limitations of this study warrant consideration. First, the cross-sectional analysis of cardiometabolic markers limits our ability to establish definitive causality regarding the transition between AF phenotypes. Second, clinically inferred MASLD is defined by clinical and non-invasive indices; no liver ultrasound, transient elastography, controlled attenuation parameter, or histological confirmation was performed, and liver biopsy remains the diagnostic gold standard despite its recognized limitations [19]. Third, FIB-4—a non-invasive proxy of hepatic fibrosis derived from age, transaminases, and platelet count—was not used in this cohort as a standalone biomarker of histologically confirmed hepatic fibrosis. Given that the PermAF group was substantially older (median 79.5 years) and carried a markedly higher burden of chronic heart failure (78.6%), chronic kidney disease (71.4%), and polypharmacy (statins, diuretics, SGLT2 inhibitors—80%, 69%, and 65%, respectively), the elevated FIB-4 values observed in this subgroup should be interpreted primarily as a composite marker of age and cardio-renal comorbidity, rather than as direct evidence of hepatic fibrosis [13,16,34]. Fourth, the definition and phenotype stratification of CHF may have led to overlap between “CHF” as a clinical diagnosis and the HFpEF/HFmrEF/HFrEF categories, particularly in subgroups with EF values close to the cut-offs. Fifth, the observed reductions in lipid levels and MASLD prevalence in advanced AF (“metabolic paradox”) may be partially influenced by statin therapy, diuretics, SGLT2 inhibitors, and the metabolic demands of advanced heart failure, which were not uniformly controlled for across subgroups [10]. Sixth, although propensity-score matching was applied with pre-specified covariates and the final matched sample achieved good balance (maximal SMD ≈ 0.13), residual confounding cannot be fully excluded, and the moderate matched sample size may limit statistical power. Finally, the single-center design and the cross-sectional AF subtype classification mean that the proposed metabolic-to-cardio-renal transition should not be interpreted as a universal longitudinal trajectory unless supported by dedicated prospective data. Furthermore, both the TG/HDL cholesterol ratio and the circulating profile of inflammatory and fibrotic biomarkers—recently highlighted as clinically informative across the metabolic syndrome–AF continuum [29,31] were not prospectively collected in our protocol and should be incorporated into future pre-specified substudies of this cohort.

## 5. Conclusions

In this prospective, single-center cohort, clinically inferred MASLD was not independently associated with the presence or severity of AF after rigorous adjustment for cardiometabolic, renal, and treatment-related confounders. Nevertheless, AF subtypes exhibited clinically meaningful cardiometabolic heterogeneity: PaAF showed a predominantly metabolic character with the highest MASLD prevalence and TG/HDL ratio, whereas permanent AF was dominated by cardio-renal impairment and elevated FIB-4 values. Importantly, the elevated FIB-4 observed in our permanent AF subgroup should not be interpreted as direct evidence of histologically confirmed hepatic fibrosis, but as a composite marker that most plausibly reflects age and the cardio-renal burden co-impairing this subgroup.

The apparent “metabolic paradox” in advanced AF most likely reflects a stage-dependent phenotypic transition from modifiable metabolic drivers toward irreversible cardio-renal and fibrotic remodeling, modulated by pharmacotherapy, age, and the systemic consequences of advanced comorbidity—rather than a true inverse biological relationship between MASLD and AF.

These findings relate to clinically inferred MASLD defined by clinical and non-invasive indices and highlight the need for subtype-specific risk stratification, image- and histology-based MASLD assessment in AF populations, and caution when interpreting FIB-4 as a marker of hepatic fibrosis in the elderly.

## Figures and Tables

**Table 1:** Comparison of demographic characteristics, clinical parameters, comorbidity profile, and pharmacotherapy between the control group and patients with AF.

Parameter	Control Group (*n* = 119)	AF Group (*n* = 208)	*p*-Value	|SMD|
Comorbidity:				
CAD [%]	26.05	31.73	0.279 ***	
CHF [%]	31.9	61.06	**<0.001** ***	
HFrEF [%]	20.17	17.31	0.599 ***	
HFmrEF [%]	7.56	17.31	**0.018** ***	
HFpEF [%]	72.27	65.38	0.259 ***	
HA [%]	71.4	72.12	0.894 ***	
T2DM [%]	34.4	36.06	0.837 ***	
CKD [%]	29.4	51.92	**<0.001** ***	
MASLD [%]	43.7	41.83	0.742 ***	
Sex [% of M]	59.66	68.75	0.096 *	0.788
Age [years]	64 [45; 72]	71.0 [30; 98]	**<0.001** *	0.148
BMI [kg/m^2^]	28.4 [24.7; 31.6]	28.4 [17.1; 45.2]	0.279 *	0.174
WC [cm]	99.8 ± 14.0	102 ± 13	0.131 **	0.146
WHR	0.94 [0.87; 1.01]	0.96 ± 0.08	0.133 *	0.788
T-CH [mg/dL]	174 [138; 208]	158 [48; 353]	**0.028** *	0.279
LDL-CH [mg/dL]	104 [76; 128]	90 [28; 236]	**0.028** *	0.332
HDL-CH [mg/dL]	40 [31; 54]	45 [14; 98]	**0.046** *	0.196
TG [mg/dL]	144 [89; 210]	115 [31; 375]	**0.001** *	0.427
TG/HDL ratio	3.33 [2.24; 5.98]	2.56 [1.60; 4.20]	0.309 *	0.424
Creatinine [µmol/L]	85 [70; 100]	95 [12; 311]	**<0.001** *	0.498
Urea [mmol/L]	6.1 [4.6; 7.4]	7.2 [3.1; 25.4]	**<0.001** *	0.546
Uric acid [µmol/L]	322 [263; 414]	344 [101; 920]	0.166 *	0.144
eGFR [ml/min/1.73 m^2^]	77 [56; 94]	59 [14; 117]	**<0.001** *	0.743
ALT [U/L]	22.9 [16.6; 34.4]	22.1 [4.0; 191.0]	0.353 *	0.109
AST [U/L]	25.7 [21.1; 32.0]	26.4 [12.5; 176.8]	0.988 *	0.028
GGTP [U/L]	29.2 [20.2; 42.8]	31.1 [5.6; 267.1]	0.078 *	0.249
FPG [mmol/L]	5.65 [5.17; 6.56]	5.69 [3.38; 12.37]	0.902 *	0.166
HbA1c [%]	5.8 [5.4; 6.4]	5.8 [4.0; 10.5]	0.755 *	0.073
EF [%]	56 [47; 61]	54 [7; 75]	0.248 *	0.055
HIS	36.7 ± 6.5	36.9 ± 5.7	0.858 **	0.020
FLI	66 [36; 85]	64 [4; 99]	0.492 *	0.127
FIB-4	1.56 [0.97; 2.16]	1.92 [0.48; 10.03]	**<0.001** *	0.446

AF—atrial fibrillation; ALT—alanine aminotransferase; AST—aspartate aminotransferase; BMI—body mass index; CAD—coronary artery disease; CHF—chronic heart failure; CKD—chronic kidney disease; EF—ejection fraction; eGFR—estimated glomerular filtration rate; FIB-4—Fibrosis 4 Index; FLI—Fatty Liver Index; FPG—fasting plasma glucose; GGTP—gamma-glutamyltransferase; HA—hypertension; HbA1c—glycated hemoglobin; HFmrEF—heart failure with mildly reduced ejection fraction; HFpEF—heart failure with preserved ejection fraction; HFrEF—heart failure with reduced ejection fraction; HDL-CH—HDL cholesterol; LDL-CH—LDL cholesterol; MASLD—clinically inferred metabolic dysfunction-associated steatotic liver disease; T-CH—total cholesterol; T2DM—type 2 diabetes mellitus; TG—triglycerides; WC—waist circumference; WHR—waist to hip ratio. |SMD|—standardized mean reported as absolute values. The *p*-value was assessed using the Mann–Whitney U test (*), Student’s *t*-test (**), or Fisher’s Exact Test (***). Data are expressed as percentages, medians (quartile 1; quartile 3), or as mean ± SD. Bolded results indicate statistically significant differences.

**Table 2:** Comparison of demographic characteristics, clinical parameters, comorbidity profile, and pharmacotherapy among the control group and AF subgroups.

	Control Group (*n* = 119)	PAF (*n* = 68)	PeAF (*n* = 70)	PermAF (*n* = 70)	*p*-Value	Max |SMD|
Comorbidity:						
CAD [%]	26.0	23.5	31.4	40.0	0.129 ^CH^	-
CHF [%]	31.9	42.6	61.4	78.6	**<0.001 ^CH^**	-
HFrEF [%]	19.33	19.12	20	8.57	**0.034 ^CH^**	-
HFmrEF [%]	4.2	8.82	18.57	21.43	**0.001 ^CH^**	-
HFpEF [%]	8.4	14.71	21.43	48.57	**<0.001 ^CH^**	-
HA [%]	71.4	70.6	78.6	67.1	0.494 ^CH^	-
T2DM [%]	34.4	38.2	35.7	32.8	0.923 ^CH^	
CKD [%]	29.4	35.3	50.0	71.4	**<0.001 ^CH^**	
MASLD [%]	43.7	47.1	51.4	27.1	**0.021 ^CH^**	
Sex (males, %)	59.66	75	71.42	60	NS ^A^	-
Age [years]	64 [45; 72]	65.0 [56.5; 72.0]	70.0 [65; 75] *	79.5 [73; 85] *^#$^	**<0.001 ^KW^**	1.3971
BMI [kg/m^2^]	28.4 [24.7; 31.6]	28.4 [26.3; 31.9]	28.8 [26.5; 31.5]	27.6 [25.2; 31.0]	NS ^A^	0.2609
WC [cm]	99.8 ± 14.0	102 ± 13	103 ± 11	100 ± 13	NS ^A^	0.2820
WHR	0.94 [0.87; 1.01]	0.96 [0.90; 1.00]	0.97 [0.91; 1.02]	0.96 [0.90; 1.02]	NS ^A^	0.2362
T-CH [mg/dL]	174 [138; 208]	174 [139; 204]	163 [126; 199] *	146 [123; 181] ^#^	**0.003** ^A^	0.5109
LDL-CH [mg/dL]	104 [76; 128]	95 [75; 116]	90 [69; 120]	89 [68; 110]	NS ^KW^	0.3784
HDL-CH [mg/dL]	40 [31; 54]	43 [35; 54]	42 [34;56]	46 [39; 57]	NS ^A^	0.3632
TG [mg/dL]	144 [89; 210]	133 [88; 187]	118 [90; 166]	88 [67; 118] *^#$^	**0.001 ^KW^**	0.6775
TG/HDL ratio	3.32 [2.24; 5.98]	3.04 [2.24; 4.79]	3.11 [1.80; 4.65]	1.93 [1.30; 2.79] *^#$^	**<0.001 ^KW^**	0.6834
Creatinine [µmol/L]	85 [70; 100]	92 [80; 110]	96 [82; 117] *	97 [80; 138] *	**<0.001 ^KW^**	**0.6913**
Urea [mmol/L]	6.1 [4.6; 7.4]	6.2 [5.4; 8.1]	7.1 [5.4; 9.3] *	8.5 [6.3; 11.4] *^#^	**<0.001 ^KW^**	**0.9721**
Uric acid [µmol/L]	322 [263; 414]	340 [300; 404]	349 [299; 399]	355 [284; 444]	NS ^A^	**0.2474**
eGFR [ml/min/1.73 m^2^]	77 [56; 94]	68 [54; 90]	59 [48; 71] *	51 [35; 60] *	**<0.001 ^KW^**	**1.1918**
ALT [U/L]	22.9 [16.6; 34.4]	23.6 [18.4; 30.4]	23.2 [18.7; 35.4]	18.8 [13; 27] ^$^	**0.029** ^A^	**0.4318**
AST [U/L]	25.7 [21.1; 32.0]	24.6 [21.7; 31.2]	26.7 [21.8; 35.0]	26.7 [21; 32]	NS ^A^	**0.3533**
GGTP [U/L]	29.2 [20.2; 42.8]	33.3 [23.5; 47.3]	32.2 [20.4; 57.0]	30.1 [20; 55]	NS ^KW^	**0.3559**
FPG [mmol/L]	5.65 [5.17; 6.56]	5.68 [5.24; 6.26]	5.74 [5.33; 6.39]	5.61 [5.24; 6.42]	NS ^KW^	0.1951
HBA1c [%]	5.8 [5.4; 6.4]	5.7 [5.5; 6.3]	5.8 [5.6; 6.1]	5.9 [5.6; 6.3]	NS ^A^	0.1088
EF [%]	56 [47; 61]	56 [45; 62]	51 [43; 58]	54 [46; 60]	NS ^A^	0.2110
HSI	36.5 [31.6; 41.1]	37.4 [34.2; 42.1]	36.5 [34.8; 39.3]	33.8 [31.0; 38.9] ^#$^	**0.021** ^A^	**0.5208**
FL1	66 [36; 85]	66 [52; 87]	72 [53; 87]	54 [33; 79]	NS ^KW^	**0.4629**
FIB-4	1.56 [0.97; 2.16]	1.61 [1.16; 2.04]	1.74 [1.35; 2.27]	2.67 [2.0; 3.6] *^#$^	**<0.001 ^KW^**	**1.0233**

ALT—alanine aminotransferase; AST—aspartate aminotransferase; BMI—body mass index; CAD—coronary artery disease; CKD—chronic kidney disease; CHF—chronic heart failure; EF—ejection fraction; eGFR—estimated glomerular filtration rate; FIB-4—Fibrosis 4 Index; FPG—fasting plasma glucose; GGTP—gamma-glutamyltransferase; HA—hypertension; HFmrEF—heart failure with mildly reduced ejection fraction; HFpEF—heart failure with preserved ejection fraction; HFrEF—heart failure with reduced ejection fraction; HSI—Hepatic Steatosis Index; HDL-CH—HDL cholesterol; LDL-CH—LDL cholesterol; MASLD—clinically inferred metabolic dysfunction-associated steatotic liver disease; PAF—paroxysmal atrial fibrillation; PeAF—persistent atrial fibrillation; PermAF—permanent atrial fibrillation; T2DM—type 2 diabetes mellitus; T-CH—total cholesterol; TG—triglycerides; WC—waist circumference; WHR—waist to hip ratio. Max |SMD| absolute values of the maximum standardized mean between compared groups. *p*-value was assessed using the Chi-square test (^CH^) for comorbidities (data are expressed as percentages) and using one-way ANOVA followed by Tukey’s post hoc test for unequal sample sizes (^A^) or using Kruskal–Wallis followed by Dunn’s multiple comparison tests (^KW^). NS—indicates a non-significant difference between groups. * analysis vs. control group. ^#^ analysis vs. PAF. ^$^ analysis vs. PeAF. Data are expressed as medians (quartile 1, quartile 3) or mean ± SD. Bolded results indicate statistically significant differences.

**Table 3:** Prevalence of clinically inferred MASLD in patients at increased risk of atrial fibrillation compared with controls.

Control Group (*n* = 119)			AF + Group (*n* = 208)			*p*-Value
	MASLD + (*n* = 52)	MASLD − (*n* = 67)		MASLD + (*n* = 87)	MASLD − (*n* = 121)	
Sex M (*n* = 71)	52.1	47.9	Sex M (*n* = 143)	41.9	58.1	0.104
Sex F (*n* = 48)	31.3	68.7	Sex F (*n* = 65)	41.5	58.5	0.178
CAD (*n* = 31)	51.6	48.4	CAD (*n* = 66)	42.4	57.6	0.264
CHF (*n* = 38)	60.5	39.5	CHF (*n* = 127)	43.3	56.7	**0.046**
HFrEF (*n* = 23)	65.22	34.78	HFrEF (*n* = 34)	16.09	16.53	0.09
HFmrEF (*n* = 5)	60	40	HFmrEF (*n* = 34)	17.24	15.70	**0.001**
HFpEF (*n* = 10)	50	50	HFpEF (*n* = 59)	29.89	27.27	**<0.001**
HA (*n* = 85)	54.1	45.9	HA (*n* = 150)	46.0	54.0	0.144
T2DM (*n* = 41)	73.2	26.8	T2DM (*n* = 74)	48.7	51.3	**0.008**
CKD (*n* = 35)	45.7	54.3	CKD (*n* = 109)	28.4	71.6	0.065
EF ≤ 50% (*n* = 41)	53.6	46.3	EF ≤ 50% (*n* = 81)	44.4	55.56	0.220
BMI ≥ 30 kg/m^2^ (*n* = 44)	81.8	18.2	BMI ≥ 30 kg/m^2^ (*n* = 75)	70.7	29.3	0.127

AF—atrial fibrillation; BMI—body mass index; CAD—coronary artery disease; CKD—chronic kidney disease; CHF—heart failure; HA—hypertension; HFmrEF—heart failure with mildly reduced ejection fraction; HFpEF—heart failure with preserved ejection fraction; HFrEF—heart failure with reduced ejection fraction; MASLD—clinically inferred metabolic dysfunction-associated steatotic liver disease; T2DM—type 2 diabetes mellitus. *p*-value was calculated using Fisher’s exact test. Data are expressed as percentages. Bolded results indicate statistically significant differences.

**Table 4:** Prevalence of clinically inferred metabolic dysfunction-associated steatotic liver disease in patients at increased risk of atrial fibrillation stratified by AF subtype.

Control Group (*n* = 119)		PAF (*n* = 68)		PeAF (*n* = 70)		PermAF (*n* = 70)		*p*-Value
	MASLD + Group		MASLD + Group		MASLD + Group		MASLD + Group	
Sex M (*n* = 71)	52.1	Sex M (*n* = 51)	43.1	Sex M (*n* = 50)	58.0	Sex M (*n* = 42)	21.4	**0.002**
Sex F (*n* = 48)	31.3	Sex F (*n* = 17)	58.8	Sex F (*n* = 20)	35.0	Sex F (*n* = 28)	35.7	0.240
CAD (*n* = 31)	51.6	CAD (*n* = 16)	43.7	CAD (*n* = 22)	50.0	CAD *n* = 28)	35.7	0.626
CHF (*n* = 38)	60.5	CHF (*n* = 29)	51.7	CHF (*n* = 43)	53.5	CHF (*n* = 55)	30.9	**0.023**
HFrEF (*n* = 23)	65.22	HFrEF (*n* = 13)	38.46	HFrEF	46.67	HFrEF	33.33	**0.044**
HFmrEF (*n* = 5)	60	HFmrEF (*n* = 6)	33.33	HFmrEF (*n* = 13)	61.54	HFmrEF	35.71	0.45
HFpEF (*n* = 10)	50	HFpEF (*n* = 10)	80	HFpEF (*n* = 15)	53.33	HFpEF	28.57	0.28
HA (*n* = 85)	54.1	HA (*n* = 48)	52.1	HA (*n* = 55)	52.7	HA (*n* = 47)	31.9	0.076
T2DM (*n* = 41)	73.2	T2DM (*n* = 26)	53.8	T2DM (*n* = 25)	52.0	T2DM (*n* = 23)	39.1	0.051
CKD (*n* = 35)	45.7	CKD (*n* = 24)	33.3	CKD (*n* = 35)	34.3	CKD (*n* = 50)	22.0	0.148
EF ≤ 50% (*n* = 41)	53.6	EF ≤ 50% (*n* = 22)	40.9	EF ≤ 50% (*n* = 34)	50.0	EF ≤ 50% (*n* = 25)	40.0	0.646
BMI ≥ 30 kg/m^2^ (*n* = 44)	81.8	BMI ≥ 30 kg/m^2^ (*n* = 24)	83.3	BMI ≥ 30 kg/m^2^ (*n* = 29)	72.4	BMI ≥ 30 kg/m^2^ (*n* = 22)	54.5	0.073

BMI—body mass index; CAD—coronary artery disease; CHF—heart failure; CKD—chronic kidney disease; EF—ejection fraction; HA—hypertension; HFmrEF—heart failure with mildly reduced ejection fraction; HFpEF—heart failure with preserved ejection fraction; HFrEF—heart failure with reduced ejection fraction; MASLD—clinically inferred metabolic dysfunction-associated steatotic liver disease; PAF—paroxysmal atrial fibrillation; PeAF—persistent atrial fibrillation; PermAF—permanent atrial fibrillation; T2DM—type 2 diabetes mellitus. The *p*-value was calculated using Pearson’s chi-squared test. Data are expressed as percentages. Bolded results indicate statistically significant differences.

**Table 5:** Distribution of pharmacotherapy in patients with different atrial fibrillation subtypes according to clinically inferred MASLD status.

	Control Group (*n* = 119)		PAF (*n* = 68)		PeAF (*n* = 70)		PermAF (*n* = 70)	
Type of Treatment	MASLD − Group	MASLD + Group	MASLD − Group	MASLD + Group	MASLD − Group	MASLD + Group	MASLD − Group	MASLD + Group
Sartan	9 (13%)	12 (23)	6 (17)	8 (25)	10 (29)	4 (11)	6 (16)	5 (26)
ACEi	31 (46)	35 (67)	18 (51)	21 (66)	19 (56)	22 (61)	36 (71)	10 (53)
Loop diuretic	17 (25)	28 (54)	13 (37)	10 (31)	21 (62)	17 (47)	35 (69)	12 (63)
Thiazide diuretic	4 (6)	6 (11)	5 (14)	3 (9)	3 (9)	6 (17)	9 (13)	4 (8)
Aldosterone antagonist	15 (22)	22 (42)	11 (31)	13 (41)	14 (41)	17 (47)	21 (41)	9 (47)
Statin	33 (49)	38 (73)	25 (71)	19 (59)	28 (82)	25 (69)	41 (80)	15 (79)
Sitagliptin	0	1 (2)	0	0	0	1 (3)	0	1 (5)
Ezetimibe	4 (6)	6 (12)	1 (3)	2 (6)	1 (3)	5 (14)	1 (2)	2 (11)
Fibrate	2 (3)	3 (6)	1 (3)	1 (3)	2 (6)	0	0	0
Metformin	12 (18)	23 (44)	7 (20)	10 (31)	9 (26)	11 (31)	6 (12)	5 (26)
SGLT2i	15 (22)	24 (46)	13 (37)	8 (25)	18 (53)	16 (44)	33 (65)	11 (58)
SU	1 (1)	2 (4)	1 (3)	2 (6)	3 (9)	4 (11)	2 (4)	0 (0)
Incretin mimetic	1 (1)	2 (4)	0	0	0	0	0	0
Insulin therapy	3 (4)	6 (12)	1 (3)	1 (3)	2 (6)	1 (3)	2 (4)	1 (5)

ACEi—angiotensin-converting enzyme inhibitor; MASLD—clinically inferred metabolic dysfunction-associated steatotic liver disease; PAF—paroxysmal atrial fibrillation; PeAF—persistent atrial fibrillation; PermAF—permanent atrial fibrillation; SGLT2i—sodium-glucose cotransporter 2 inhibitor. Data are presented as a number (percentage).

## Data Availability

The datasets used and/or analyzed during the current study are available from the corresponding author on reasonable request.

## Abbreviations

The following abbreviations are used in this manuscript:

AF	Atrial fibrillation
ALT	Alanine aminotransferase
AST	Aspartate aminotransferase
BMI	Body mass index
CAD	Coronary artery disease
CHF	Chronic heart failure
CKD	Chronic kidney disease
ECG	Electrocardiography
EF	Left ventricular ejection fraction
eGFR	Estimated glomerular filtration rate
FIB-4	Fibrosis-4 Index
FLI	Fatty Liver Index
FPG	Fasting plasma glucose
HA	Hypertension
HbA1c	Glycated hemoglobin
HC	Hip circumference
HDL-CH	HDL cholesterol
HSI	Hepatic Steatosis Index
GGTP	Gamma-glutamyltransferase
MASLD	Metabolic dysfunction-associated steatotic liver disease
MDRD	Modification of diet in renal disease
LDL-CH	LDL cholesterol
PAF	Paroxysmal atrial fibrillation
PeAF	Persistent atrial fibrillation
PermAF	Permanent atrial fibrillation
T2DM	Type 2 diabetes mellitus
T-CH	Total cholesterol
TG	Triglycerides
WC	Waist circumference
WHR	Waist-to-hip ratio
